# Urinary Basigin/CD147 is a useful marker of acute T cell-mediated rejection in kidney transplant recipients

**DOI:** 10.1080/0886022X.2025.2479574

**Published:** 2025-03-24

**Authors:** Kenta Futamura, Makoto Tsujita, Tomoki Kosugi, Akihiro Ryuge, Manabu Okada, Takahisa Hiramitsu, Shunji Narumi, Asami Takeda, Yoshihiko Watarai, Kunio Morozumi, Shoichi Maruyama

**Affiliations:** a Department of Kidney Disease Center, Transplant Nephrology and Surgery, Japanese Red Cross Aichi Medical Center Nagoya Daini Hospital, Nagoya, Japan; b Department of Nephrology, Masuko Memorial Hospital, Nagoya, Japan; c Department of Nephrology, Nagoya University Graduate School of Medicine, Nagoya, Japan; d Internal Medicine, Ryuge Internal Medicine Kamiotai Clinic, Nagoya, Japan

**Keywords:** Basigin/CD147, kidney transplantation, acute T cell-mediated rejection, kidney biopsy

## Abstract

**Background:**

Acute T cell-mediated rejection (ATCMR) is a severe negative outcome of kidney transplantation; however, it currently has no reliable marker in Japan.

**Methods:**

This cross-sectional study was conducted at the Japanese Red Cross Aichi Medical Center Nagoya Daini Hospital from 2016 to 2018 to determine whether plasma or urinary Basigin/CD147 is an effective marker of ATCMR. Plasma and urinary samples were obtained when episode graft biopsies were performed.

**Results:**

Forty-six kidney transplant recipients received graft biopsies. Three of them missed plasma and urinary samples and three in ATCMR were on postrejection treatment. Graft biopsy results revealed ATCMR in 12 of them, calcineurin inhibitor nephrotoxicity (CIN) in nine, chronic active antibody-mediated rejection (CAAMR) in nine, BK nephropathy, recurrence IgA nephropathy, necrotic glomerulonephritis, and infection-related glomerulonephritis in one each, and other complications in six. The urinary Basigin/CD147 levels of patients in the ATCMR group [759.4 (490.0, 843.0)] pg/gCre were significantly higher than the levels of patients in the CAAMR [247.0 (157.1, 288.8)] and CIN groups [379.1 (264.7, 456.7)] pg/gCre (*p* < 0.001). No statistical difference in plasma Basigin/CD147 levels was observed between those groups. At a urinary Basigin/CD147 of 631.5 µg/gCre, 75% sensitivity and 84% specificity with an area under the curve of 0.80 were attained for the diagnosis of graft rejection.

**Conclusion:**

Urinary Basigin/CD147 may be a potential marker for ATCMR in kidney transplant recipients. Further studies will be needed to clarify the effectiveness of Basigin/CD147.

## Introduction

Acute T cell-mediated rejection (ATCMR) is a severe complication of kidney transplantation and is marked by lymphocytic infiltration of the tubules, interstitium, and, in some cases, the arterial intima, causing interstitial fibrosis and tubular atrophy and resulting in poor graft survival [1]. Early diagnosis is critical to preventing graft function deterioration. Presently, graft biopsy remains the best way to diagnose; however, it is invasive and cannot be performed regularly. Consequently, some biomarkers for rejection that are alternatives for graft biopsy have been reported [2–4]. Contemporary biomarker research for ATCMR has focused on regulatory T cells (Tregs) and their associated genes, such as *FOXP3* and *CTLA-4*. However, these biomarkers have limitations in terms of sensitivity and specificity, and their expression patterns can be influenced by various factors, including immunosuppressive medications. To the best of our knowledge, a reliable, noninvasive biomarker for ATCMR has not yet been established yet [5].

Basigin/CD147 is a transmembrane glycoprotein categorized within the immunoglobulin superfamily, which contributes to cell survival, migration, and cancer invasion and is widely expressed in different tissues. It has been implicated in various pathological conditions, such as cancer, inflammation, and T cells. Basigin/CD147 performs pleiotropic functions by binding to monocarboxylate transporters, which play important roles in glycolysis regulation. T cells require glycolysis for differentiation, proliferation, and activation, which is regulated by Basigin/CD147 [6]. In normal kidneys, high Basigin/CD147 expression is identified only in the basolateral side of the tubular epithelial cells (TECs) [7]. Mori et al. reported that the assessment of plasma and urinary Basigin/CD147 levels might help elucidate the activity of different kidney diseases [8].

In the field of kidney transplantation, Nalewajska et al. reported that Basigin/CD147 was associated with long-term graft survival [9]. However, the role of Basigin/CD147 in ATCMR remains unknown in clinical application. Interleukin-17 (IL-17), generated by T helper 17 (Th17), plays an essential role in renal allograft rejection [10]. IL-17 induces several cytokines and inflammatory mediators. A previous report revealed a notable increase in IL-17 protein expression within the infiltrating cells on TECs in ATCMR [11]. Maeda et al. demonstrated that Basigin/CD147 plays an important role in Th17 cell differentiation as a negative regulator by suppressing the Interleukin-6 (IL-6)/signal transducer and activator of transcription 3 pathway [12].

These findings allowed us to delve into the fundamental question regarding the correlation of Basigin/CD147 with ATCMR in kidney transplant recipients. This study aimed to determine whether Basigin/CD147 was a useful marker of ATCMR.

## Materials and methods

### Study design

We conducted a single-center cross-sectional study to assess Basigin/CD147 as a useful marker of ATCMR. From 2016 to 2018, eligible kidney transplant recipients taking episode graft biopsies were enrolled at the Japanese Red Cross Aichi Medical Center Nagoya Daini Hospital. The criteria for episode graft biopsy were as follows: (1) 30% increase in the plasma creatinine (Cre) level from baseline, (2) *de novo* donor-specific antibody positivity, (3) urinary protein ≥0.5 g/gCre, or (4) new-onset hematuria. They had undergone kidney transplantation more than two months before and were aged >20 years. Plasma and urinary samples to measure Basigin/CD147 were obtained when the biopsy was performed. Patients were recommended to drink more than two liters of water daily to avoid dehydration. Plasma and spot urinary samples were centrifuged at 2000×*g* for 5 min to eliminate cellular components and debris, after which equal volumes of supernatants were stored at −80 °C. Pathological diagnosis per the 2019 Banff classification was performed by a specially trained nephrologist (A.T.) as shown in Table 1.

**Table 1.** Pathological diagnosis in this study (*n*).

Acute T cell-mediated rejection (ATCMR)	
IA	8
IB	3
IIB	1
Total	12
Calcineurin inhibitor nephrotoxicity (CNIN)	9
Chronic active antibody-mediated rejection (CAAMR)	9
BK nephropathy	1
Recurrence IgA nephropathy	1
Necrotic glomerulonephritis	1
Infection-related glomerulonephritis	1
Non-specific findings	6

### Measurements

Plasma and urinary CD147 levels were quantified using commercial enzyme-linked immunosorbent assay kits per the manufacturer’s instructions (R&D Systems, Minneapolis, MN). Both CV intra- and inter-assay of this assay were less than 10%. Plasma and urinary Basigin/CD147 levels were measured using Thermo Scientific Multiskan Sky.

### Participants

**Figure 1.**
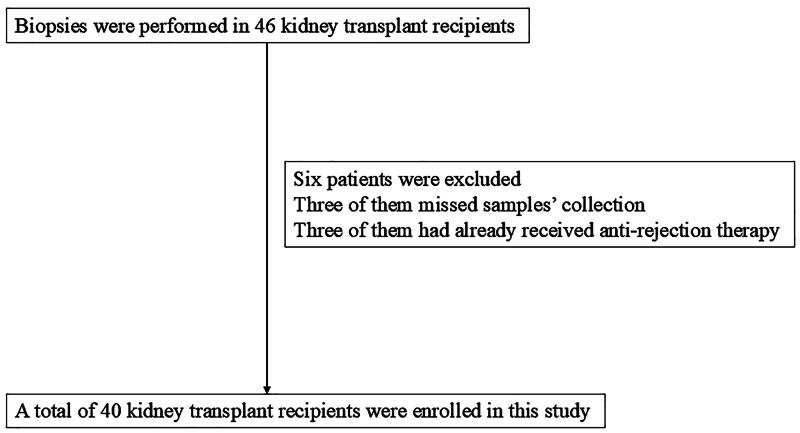
Flowchart of the study procedure.

Episode kidney biopsies were performed on 46 patients in this study. Because three of them missed sample collection and three of them had already received antirejection treatment before the biopsy, six patients were omitted from this study (Figure 1). Based on graft biopsies, 12 of them were categorized into the ATCMR group, nine into the calcineurin inhibitor nephrotoxicity (CIN) group, and nine into the chronic active antibody-mediated rejection (CAAMR) group.

### Statistical analyses

Continuous variables are expressed as the mean ± standard deviation (SD) for normally distributed data or the median (interquartile range) for non-normally distributed data. Categorical data are presented as frequencies and percentages. Between-group differences were assessed using the Krusukal–Wallis test or the Steel–Dwass test. Receiver operating characteristics were performed to determine the cutoff value of urinary Basigin/CD147 in the diagnosis of ATCMR. All *p-*values were two-sided. A *p-*value of <0.05 was considered statistically significant. Statistical analyses were performed using JMP Version 17 (SAS Institute, Inc., Cary, NC, USA).

## Results

The characteristics of our study participants are shown in Table 2. Graft biopsy findings revealed ATCMR in 12 of them, CIN in nine, CAAMR in nine, BK nephropathy, recurrent IgA nephropathy, necrotic glomerulonephritis, and infection-related glomerulonephritis in one each, and other complications in six.

**Table 2.** Patients’ characteristics in this study.

Variables	Overall (*n* = 40)	CNI (*n* = 9)	ATCMR (*n* = 12)	CAAMR (*n* = 9)	*p* value
Age (year)	51.5 ± 14.8	53.9 ± 16.9	55.4 ± 15.3	46.9 ± 13.8	0.423
Sex (male), *n* (%)	28 (70.0%)	5 (55.6%)	8 (66.7%)	7 (77.8%)	0.774
Body mass index (kg/m^2^)	23.7 ± 3.6	24.0 ± 2.3	23.9 ± 4.8	25.2 ± 2.6	0.683
Duration after kidney transplantation (month)	24 (5, 50)	7 (2.5, 72)	5.5 (2.3, 50)	36 (13, 73.5)	0.551
eGFRcre (mL/min/1.73m^2^)	29.8 ± 9.8	26.4 ± 5.2	25.6 ± 11.2	34.4 ± 9.0	0.084
Systoric blood pressure (mmHg)	131.5 ± 11.7	129.2 ± 13.1	134.5 ± 10.3	125.6 ± 12.9	0.245
Diastoric blood pressure (mmHg)	79.9 ± 8.6	81.4 ± 12.2	81.4 ± 8.2	75.6 ± 7.4	0.303
Serum Basigin (pg/mL)	4,608 (2259, 5149)	2,384 (1160, 5228)	5,079 (4189, 5870)	4,188 (2527, 5370)	0.128
Urinary Basigin (µg/gCre)	411.9 (249.6, 648.9)	379.1 (264.7, 456.7)	759.4 (490.0, 843.0)	247.0 (157.1, 288.8)	< 0.001
Proteinuria (g/gCre)	0.26 (0.10, 0.42)	0.26 (0.11, 0.57)	0.34 (0.19, 0.45)	0.09 (0.05, 0.27)	0.232
Immunosuppresive drug					
Tacrolimus use, *n* (%)	21 (52.5%)	3 (33.3%)	5 (41.7%)	3 (33.3%)	0.331
Cycrosporine use, *n* (%)	19 (47.5%)	6 (66.7%)	7 (58.3%)	6 (66.7%)	
Mycophenolate Mofetil use, *n* (%)	40 (100%)	9 (100%)	12 (100%)	9 (100%)	
Prednisolone use, *n* (%)	40 (100%)	9 (100%)	12 (100%)	9 (100%)	
Complication					
Hypertension, *n* (%)	30 (75.0%)	5 (62.5%)	7 (58.3%)	8 (88.9%)	0.292
Hyperlipidemia, *n* (%)	28 (70.0%)	4 (50.0%)	9 (75.0%)	6 (66.7%)	0.513
Diabetes mellitus, *n* (%)	5 (12.5%)	1 (11.1%)	2 (16.7%)	2 (22.2%)	0.816

Data are expressed as means ± standard deviation, median (interquartile range), or number.

### Plasma and urinary CD147 levels

**Figure 2.**
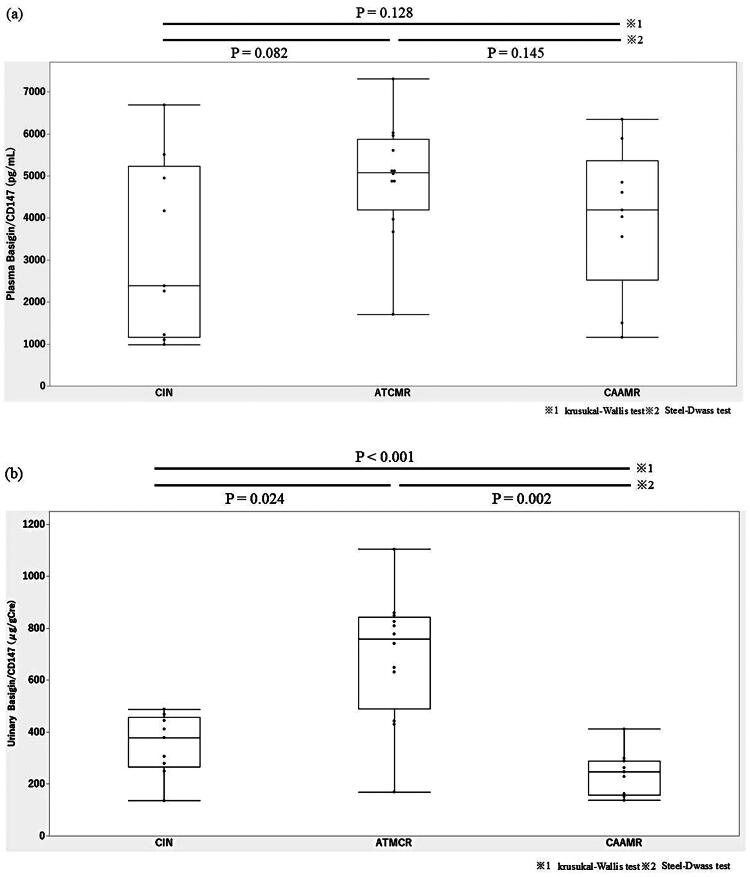
(a) Plasma Basigin/CD147 levels of patients in the CIN, ATCMR, and CAAMR groups. (b) Urinary Basigin/CD147 levels of patients in the CIN, ATCMR, and CAAMR groups. CIN: calcineurin inhibitor nephrotoxicity; ATCMR: acute T cell-mediated rejection; CAAMR: chronic active antibody-mediated rejection.

**Figure 3.**
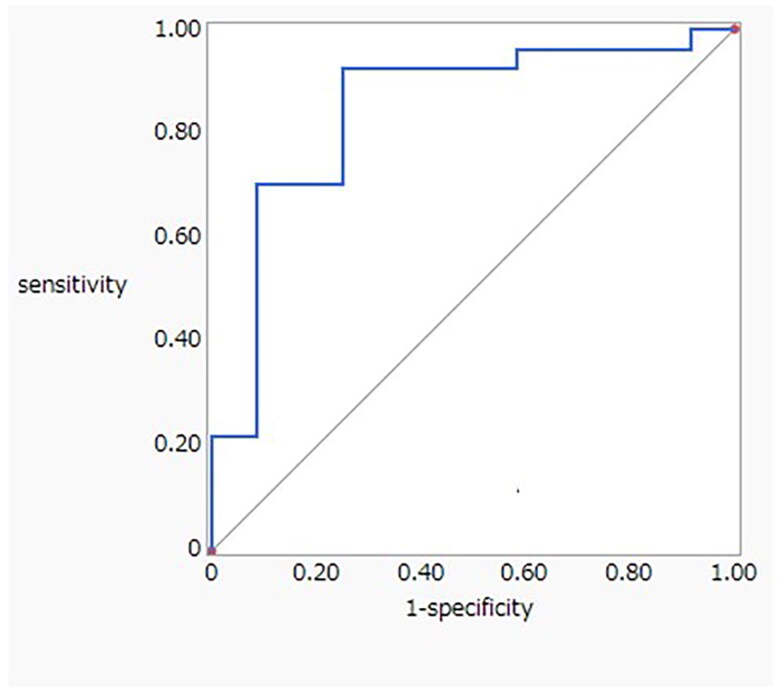
Receiver operating characteristic curve to evaluate the accuracy of urinary Basigin/CD147 in diagnosing T cell-mediated rejection.

The median plasma CD147 levels were [4608 (2259, 5149)] pg/mL overall, [5079 (4189, 5870)] pg/mL in the ATCMR group, [4188 (2527, 5370)] pg/mL in the CAAMR group, [2384 (1160, 5228)] pg/mL in the CIN group. No statistical difference in plasma Basigin/CD147 levels was observed between those groups. The median urinary Basigin/CD147 µg/gCre levels were [411.9 (249.6, 648.9)] µg/gCre. Figure 2 illustrates that urinary Basigin/CD147 levels of patients in the ATCMR group [759.4 (490.0, 843.0)] µg/gCre were significantly higher than those of patients in the CAAMR group [247.0 (157.1, 288.8)] µg/gCre and CIN groups [379.1 (264.7, 456.7)] µg/gCre (*p* < 0.001). At a urinary Basigin/CD147 of 631.5 µg/gCre, 75% sensitivity and 84% specificity, were achieved in the diagnosis of an ATCMR with an area under the curve (AUC) of 0.80 (Figure 3). Conversely, at a plasma Basigin/CD147 of 4898 pg/mL, 50% sensitivity and 68% specificity, were achieved for the diagnosis of ATCMR with a AUC of 0.63.

Table 3 shows factors associated with urinary Basigin/CD147. Age, eGFR, and different immunosuppressive drugs had less influence on it.

**Table 3.** Associations of risk factors with urinary Basigin/CD147 (*n* = 40).

	Univariate			Multivariate		
	β	t	*p*	β	t	*p*
Age	0.29	1.82	0.076	0.16	1.12	0.27
eGFR	−0.32	−2.04	0.049	−0.16	−1.19	0.24
Cyclosporine use	0.08	0.47	0.639	0.07	0.49	0.62
ATCMR	0.59	4.5	< 0.001	0.53	3.73	< 0.001

ATCMR: acute T cell-mediated rejection.

As for cases not included in the three groups, the plasma and urinary Basigin/CD147 levels were 3608 pg/mL and 617.1 µg/gCre, respectively, in the patient with BK nephropathy, 4888 pg/mL and 304.1 µg/gCre, respectively, in the patient with recurrent IgA nephropathy, 4700 pg/mL and 922.0 µg/gCre, respectively, in the patient with necrotic glomerulonephritis, and 1746 pg/mL and 551.7 µg/gCre, respectively, in the patient with infection-related glomerulonephritis.

## Discussion

This study shows that urinary Basigin/CD147 may be an effective marker of ATCMR in kidney transplant recipients. The injury of ATCMR mainly occurs in the tubules and interstitium of the grafted kidney. It is reasonable that Basigin/CD147 expressed in the tubules and interstitium could be more detected in urine than in plasma.

Although the association of urinary Basigin/CD147 with ATCMR has not been previously reported, the reason could be explained in the previous paper. First, considering the diagnosis of ATCMR in kidney allografts, it was defined by infiltration of the interstitium by T cells and macrophages and tubulitis only in non-scarred areas of the allograft [13]. Among the CD4^+^ T cell subsets, Th17 and Tregs are differentiated from CD4^+^ T cells, and imbalanced Th17 and impaired Tregs are the primary key factors involved in the pathogenesis of ATCMR [14]. Loong et al. reported that in patients with subclinical borderline rejection, IL-17 mRNA expression in infiltrating mononuclear cells of urinary sediment and confirmed that human renal epithelial cells exposed to IL-17 produced by Th17 can generate inflammatory mediators with the potential to stimulate early alloimmune responses [15].

Regarding the association of Basigin/CD147 with kidney damage, Kato et al. revealed that Basigin/CD147 deficiency decreased inflammatory cells into the tubule-interstitium using a renal ischemia/reperfusion injury mouse model, which is characterized by a significant influx of inflammatory cells following reperfusion. They concluded that Basigin/CD147 might play a crucial role in the prevention of acute kidney injury [16]. Furthermore, previous papers indicated that Basigin/CD147 was involved with transplant rejection. Lean et al. performed allogeneic skin transplantation in a mouse model to assess whether the blockade of CD147 can suppress the rejection reaction and to determine whether CD147 antibodies could be created as specific immunosuppressors for the graft rejection response. They verified that CD147 blockade prolonged the survival of transplanted skin and lowered the level of inflammatory cell infiltration in transplanted skin by reducing the serum levels of IL-17 generated by Th17 and the proportions of peripheral blood CD4+ and CD8+ memory T cells, and CD147 antibodies have the potential to develop into new target-specific immunosuppressant drugs [17]. Furthermore, Okubo et al. investigated whether CD147 is involved in the pathogenesis of Th17-cell-mediated immune disorders and analyzed its impact on the development of psoriasis. Per their findings, naïve CD4+ T cells from CD147−/− mice demonstrated low potential for differentiation into Th17 cells, in response to stimulation with IL-6 and transforming growth factor β, and that CD147 modulates the differentiation of CD4+ T cells into Th17 cells and concluded that CD147 was crucial for the development of psoriasis by inducing Th17 cell differentiation [18]. This paper demonstrated that Basigin/CD147 plays an important role in CD4+ T cell differentiation and ATCMR *via* Th17 differentiation.

Even during the maintenance period, ATCMR is one of the severe complications associated with a poor graft prognosis. Early detection is important to help prevent poor prognoses. In clinical practice, ATCMR, CIN, and CAAMR are common causes requiring differential diagnoses. In our study, urinary Basigin/CD147 levels in the ATCMR group were significantly higher in the CIN and CAAMR groups. The underlying mechanism remains unclear; however, it was thought that urinary Basigin/CD147 levels might reflect tubulointerstitial damage because Sun et al. reported that Basigin/CD147 expression was associated with tubulointerstitial injury, not in ATCMR but in IgA nephropathy [19]. Regarding tubulointerstitial injury, it is also applicable in the pathology of BK virus nephropathy. Therefore, in this study, an increase in urinary Basigin/CD147 level was observed in BK virus nephropathy which included interstitial inflammation, and tubulitis [20]. Alternatively, no statistical difference in plasma Basigin/CD147 levels was observed between those groups. This might be because it was a small study. In terms of the effectiveness of urinary and plasma Basigin/CD147 levels, it is thought that the urinary level, with its higher AUC, may be more effective as a marker.

In two patients with glomerulitis, plasma Basigin/CD147 levels appeared to exceed the average values (4888 pg/mL in the patient with recurrent IgA nephropathy and 4700 pg/mL in the patient with necrotic glomerulonephritis). Plasma Basigin/CD147 might be a good marker for glomerular damage. However, because patients with severe glomerular damage were not included in this study, further investigation will be required to elucidate these associations in kidney transplant recipients. Furthermore, it is important to note that urinary Basigin/CD147 levels may increase in diseases associated with tubulointerstitial damage other than ATCMR, such as BK nephropathy. Urinary Basigin/CD147 should be assessed based on clinical findings. As high urinary Basigin/CD147 levels indicate tubulointerstitial damage, it shows that a kidney biopsy may not always be necessary to make a diagnosis.

Regarding a useful biomarker for ATCMR, donor-derived cell-free DNA is a promising biomarker; however, it is costly and not yet widely accessible in clinical practice [21]. Consequently, there is a critical need for a reliable and easily measurable biomarker for ATCMR. Basigin/CD147 can be detected in urine, making it a noninvasive method of diagnosing ATCMR. Early detection can lead to timely interventions that will improve the prognosis. The traditional graft function monitoring techniques often require blood tests or biopsies, which can be invasive and uncomfortable. Urinary Basigin/CD147 offers a less invasive alternative, allowing for more frequent and comfortable graft function monitoring. This may lead to better patient compliance and more accurate tracking of disease progression or response to treatment.

Nevertheless, this study had the following limitations: (1) Only a few participants were enrolled in a single center. (2) This study was cross-sectional. (3) Only Japanese were included. (4) Some potential confounders such as differences in immunosuppressive regimens or other post-transplant complications might have influenced the results. (5) The expression of Basigin/CD147 in biopsy tissue was not available. (6) Longitudinal studies to assess the temporal relationship between urinary Basigin/CD147 levels and ATCMR onset will be needed were lacking.

In conclusion, urinary Basigin/CD147 might be a potential marker for ATCMR in kidney transplant recipients. Further studies will be required to clarify the validation of Basigin/CD147.
